# Influence of Geometric Imperfections on Buckling Resistance of Reinforcing Bars during Inelastic Deformation

**DOI:** 10.3390/ma13163473

**Published:** 2020-08-06

**Authors:** Jacek Korentz

**Affiliations:** Faculty of Civil Engineering, Architecture and Environmental Engineering, University of Zielona Góra, Szafrana 1, 65-516 Zielona Góra, Poland; j.korentz@ib.uz.zgora.pl; Tel.: +48-68-3282416

**Keywords:** reinforcing bars, slenderness ratio, geometric imperfection, deformed shape, inelastic buckling, buckling resistance

## Abstract

This paper presents the results of numerical simulations on three main factors and their influence on the buckling resistance of reinforcing bars and on their behaviour in the range of postcritical deformations. These three factors are the shape of initial deformation, the amplitude of geometric imperfection and the slenderness of bars. The analysis was made of bars fixed on both sides for three initial shapes of deformation between adjacent stirrups, four amplitudes of geometric imperfections and eight bar slendernesses. The results of the numerical analyses carried out showed that the factors analysed have a very high influence on the inelastic buckling of the bars. The initial deformation shape, the radius of curvature and the slenderness of the bars have a significant influence on the buckling resistance of these bars and their longitudinal and transverse deformations. The research demonstrates that bars which are bent or compressed initially have a smaller resistance to buckling compared to straight bars, as the amplitude of geometric imperfections increases and the slenderness of the members increases. However, for the deformation shape of the bars, which is accompanied by shear forces, the drop in the buckling resistance of the members is small, and resistance to buckling for items with a small slenderness was higher than that of straight bars.

## 1. Introduction

The impact of seismic and exceptional loads on reinforced concrete structures may lead to the occurrence of postcritical states in structural elements. It is, therefore, necessary to ensure that the load-bearing capacity of the structure is maintained as long as possible after yielding. Load-bearing structures, should, as a consequence, have an adequate level of ductility. During an earthquake in reinforced concrete structures, buckling of the longitudinal reinforcing bars often occurs. The buckling of bars preceded by spalling concrete cover is accompanied by a sudden and progressive decrease in the load-bearing capacity of the reinforced concrete element. In order to avoid too-early buckling of the longitudinal reinforcement bars, the longitudinal and transverse reinforcement must be adequately arranged in the plastic hinge. In the case of the analysis of reinforced concrete elements in a state of postcritical deformations, when the limit of deformations of the cover concrete are exceeded and at the same time, the deformations of the reinforcement are large, it should be considered that the compressive reinforcement works differently than the tensile reinforcement. This difference requires the use of a corresponding reinforcement model and appropriate constitutive laws for compressed bars, which are different from those for tensile bars. The inelastic buckling of the reinforcing bar is determined by many factors. The most important of these being the slenderness of the bar and the mechanical properties of the reinforcing steel. So far, little attention has been paid to the initial deformations of the bars caused by the acting loads that accompany the compressive forces transmitted to the longitudinal reinforcement bars. This lack of attention includes deformations of the bars due to bending moment, compression forces or shear force. The aim of this paper, therefore, is to examine the influence of the initial deformation shape of the bars, the amplitude of these deformations and the bars’ slenderness on the buckling resistance of the reinforcement bars.

## 2. Literature Review

In typical situations of the design and analysis of reinforced concrete elements, according to Eurocode 2 [1], it is assumed that the reinforcement bars do not buckle during bending, compression or other load combinations. According to Eurocode 8 [2], beams and columns in seismic-resistant frames require an adequate amount of properly spaced longitudinal reinforcement in the critical regions of beams and columns. It is also necessary to use transverse stirrups or ties. These must not only provide adequate shear and adequate confinement of the concrete core, but also must, above all, prevent or delay the buckling of the longitudinal reinforcement bars. It is also assumed that in the area of plastic hinges, the buckling of the rebar can occur between two adjacent stirrups. However, experience from past earthquakes and the results of experimental studies have shown that, in practice, many forms of buckling of reinforced bars will occur. The damage that structures suffer during earthquakes is analysed in detail and, after additional experimental testing, is the basis for recommending new standard recommendations.

### 2.1. Bucking Form of the Rebar

**Figure 1:**
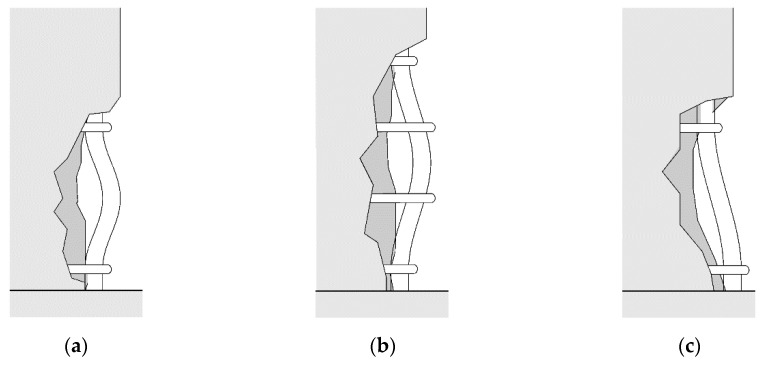
Shapes of buckled bars: (**a**) single tie interval without translation, (**b**) spanning three tie intervals and (**c**) single tie interval with translation [3].

According to Scribner [3], three basic buckling shapes of the rebar are possible (Figure 1). The rebar can buckle between two adjacent stirrups (Figure 1a) or between the first and the fourth stirrup if the ties’ resistance is insufficient (Figure 1b). When the shear deformation in the plastic hinge is significant, the buckling of the bar may be accompanied by lateral displacement of the bar nodes (Figure 1c).

Analysis of the damage to reinforced concrete wall structures during the Chilean earthquake in 2010 [4] showed that typical wall damage was concentrated at their edges. This was spalling of concrete cover and buckling of the bars over a length greater than the spacing of the transverse reinforcement (global buckling). Buckling between adjacent stirrups is local buckling and buckling longer than stirrup spacing is global buckling [5]. An analysis of the impact of the 1992–2003 [6] and 2011 [7] earthquakes in Turkey on the behaviour of reinforced concrete columns showed that local and global bar buckling was caused by poor quality concrete and errors in the longitudinal and transverse reinforcement. According to [8], seven types of column damage can be identified and the most important reason for this is inadequate transverse reinforcement. Damage to reinforced concrete columns during earthquakes in California, Japan, Turkey and Iran from 1994 to 2003 [9] was divided into two categories: bending and shear damage, and the lack of adequate ductility for bending of the columns and horizontal migration of the column nodes cause large deformations. In the research of reinforced concrete columns with different types of transverse reinforcement [10] and in the research on walls [11], several typical forms of rebar buckling were reported. According to these studies, the shapes of buckled bars in reinforced concrete columns depends on the configuration of stirrups and of their diameter and spacing, and global buckling occurs when stiff ties with small spacing are used.

### 2.2. Deformation of the Rebar

**Figure 2:**
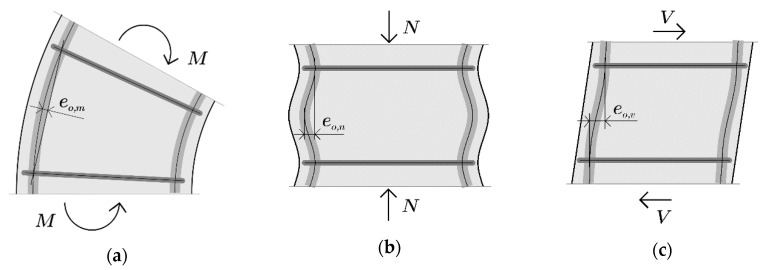
Deformation of rebar due to: (**a**) bending moment (MD), (**b**) compression force (ND) and (**c**) shear force (VD).

Figure 2 shows three basic forms of the deformation of reinforced concrete columns in a plastic hinge under the influence of acting loads. These are the deformations accompanying bending moment (MD), compression force (ND) and shear force (VD). Reinforcing bars are also subject to deformations that cause curvature of their axis and displacement of their support points with ties. In the case of bending (Figure 2a), the effect of the imposed curvature of the bar is its deflection e_o,m_, which depends on the radius of curvature of the column.

When the column is subjected to axial compression, longitudinal strain is accompanied by transverse strain (Figure 2b). The transverse strain of the column core is the smallest in the ties’ axis and the largest in the middle of the distance between the ties. As a result, the difference in transverse displacement of the longitudinal reinforcing bars is equal to e_o,n_. The difference in this strain depends on the ties’ spacing, their rigidity and load level. The shear forces induce deformations in the columns (Figure 2c), which result in the transverse displacement of bars e_o,v_ at the distance between the ties. The value of these deformations depends primarily on the shear ratio.

Research on the influence of tensile stress on the buckling of reinforcing bars in concrete columns [12] showed that with an increase in preliminary tensile deformations in the rebar, the ductility of cyclically loaded columns decreases. If high tensile deformations due to bending are present in the rebar, the buckling of the rebar occurs earlier than during axial compression after the load reversal [13,14]. This is due to the deformation of members whose radius of bending curvature decreases with increasing bending moment. Therefore, after the load is reversed, the compressive force acts on an increasing eccentricity e_o,m_ (Figure 2a). For example, for the radius of curvature of 3000 mm, the deflection of the bar over a length of 200 mm may be e_o,m_ = 1.6 mm.

During axial compression of reinforced concrete columns, there is a difference between transverse deformations at tie level and half of the distance between ties (Figure 2b). Poisson’s coefficient υ for concrete varies from 0.2 in the elastic range to 0.5 when its strength is exceeded [15,16]. However, for maximum stress, the transverse expansion of the concrete is much greater on the external surface of the specimen (υ = 1.0) than inside the specimen in its undamaged part (υ = 0.4) [17]. In the provisions of standard Eurocode 2 [1], the limit longitudinal strain of compressed concrete ε_c,u_ = 0.0035. In the case of confined concrete, its final strain ε_cc,u_ is much higher and may vary from 0.016 to 0.26 [18] and from 0.035 to 0.055 [19]. The relationship between the transverse and longitudinal deformation of the concrete after yielding also depends on the confined reinforcement and the cross-section shape of the specimen [20]. This is confirmed by Bayrak’s [21] research on the expansion of concrete core in columns, in which the lateral strain of the confined concrete decreased with an increase in the volumetric stirrup reinforcement ratio and represented 0.3 to 0.5 longitudinal strain ε_c,l_ of the tested columns. Thus, in extreme cases, the lateral strain of concrete may reach a value of ε_c,t_ = υ∙ε_c,l_ = 0.5 × 0.050 = 0.0250. On the other hand, the lateral strain of concrete core is limited by ties, whose strains are much smaller than the lateral strain of a concrete core between stirrups. When the load-bearing capacity of the reinforced concrete columns is exceeded, the ties strain ε_s,t_ may vary as follows: 0.002 [10], from 0.0015 to 0.005 [22] and 0.004 [23], and in eccentrically loaded columns, it may vary from 0.0005 to 0.00025 [24]. The lateral strain of the concrete core at tie level is the same as those of the tie. Thus, the difference in the lateral deformation of the concrete core between the ties ε_c,t_ and at ties level ε_t_, when the bearing capacity of the column is exhausted, can be quite large, e.g., ∆ε= ε_c,t_ − ε_s,t_ = 0.0250 − 0.005 = 0.02, and, therefore, the compression force of the bars will act on the eccentricity e_o,n_ (Figure 2b). In case of deformation of rebars caused by compressive force, geometric imperfections e_on_ caused by transverse permanent deformations after the unloading were analysed. Therefore, a zero-stress state in the bars was assumed for reloading.

In columns loaded cyclically, the increase in horizontal node transfers is accompanied by shear deformations. Shear induced displacements increase with the number of cycles and their amplitude, whereas bending displacements decrease with the number of cycles and their amplitude [25,26]. Therefore, the difference in horizontal displacements between the upper and lower nodes of the column can be large, i.e., up to a dozen or so centimetres, which also entails a much smaller difference in horizontal displacements of the longitudinal bars between the two adjacent stirrups (Figure 2c) and the compression force of the bar that acts on the eccentricity e_o,v_.

### 2.3. Inelastic Buckling of Reinforcing Bars

The problem of inelastic buckling of reinforcing bars has been the subject of many publications, in which the results of experimental studies [21,27,28,29,30,31] as well as the results of numerical simulations conducted [32,33,34,35] were presented. The results of numerical analyses reflect the results of experimental research well. In this research, the variability of a wide range of geometric properties of reinforcing bars was examined, while the range of the tested mechanical properties of steel was limited to a few steel grades produced in the authors’ countries. The results of these studies showed that the buckling resistance of bars depends primarily on the slenderness of the bars, expressed in stirrup spacing and their diameter, and the mechanical properties of the steel from which they are made. The buckling resistance of the bars is not lower than the yield strength if s/ϕ ≤ 20. However, for a slenderness of bars smaller than slenderness changing between 8 and 11, the buckling resistance of the bars is higher than the yield strength. For intermediate slenderness values, the buckling resistance of the bars is equal to the yield strength.

The behaviour of reinforcing bars with geometric imperfections that accompany compression deformations has been discussed in [21,27,36]. According to [21], the expansion of the concrete core causes a horizontal deflection of the bars, which contributes to a reduction of the compressive ductility of the bars and a reduction of the achievable maximum stress. According to [27], for a certain slenderness, an increase in the initial eccentricity causes a decrease in the load capacity and ductility. In [36], a model of compression reinforcing bars with geometric imperfections is proposed, which with a high degree of accordance reproduces the results of experimental studies [21].

## 3. Description of the Numerical Analyses

### 3.1. Aim and Scope of Research

The aim of the study was to determine the effect of the shape of initial deformations and their amplitude on inelastic buckling of reinforcing bars. The scope of research included three deformation shapes of bars between two adjacent stirrups (Figure 2). For these three forms of deformation, the effects of the amplitude of geometric imperfection and slenderness of the bars were investigated. Geometric imperfections were assumed to be the horizontal displacement of the central section of the bar for bending and compression deformations and the horizontal displacement of one node of the bar for shear deformations. Numerical analyses were performed for eight different bars slendernesses and for four amplitudes of imperfections. The buckling of straight bars was also analysed for eight slendernesses.

### 3.2. Reinforcing Bars Models

**Figure 3:**
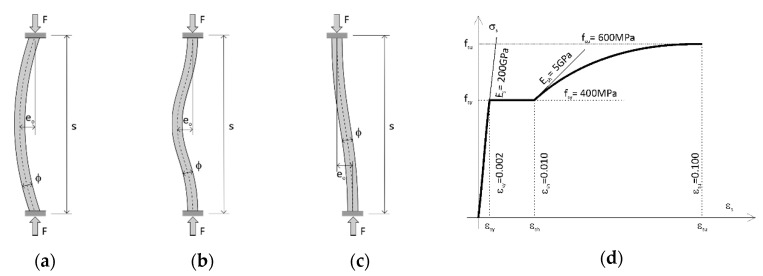
Reinforcing bars models for: (**a**) bending deformation (MD), (**b**) compression deformation (ND), (**c**) shear deformation (VD) and (**d**) material curve.

The analysis was made of bars fixed on both sides for three initial shapes of deformation between adjacent ties (Figure 3a–c), four amplitudes of geometric imperfections e_o_/ϕ and eight bar slendernesses s/ϕ. The diameter of the bars was constant ϕ = 16 mm. The shape of the bars’ deformed axis corresponded to the cosine function. The measure of the amplitude of imperfections was the relative displacement e_o_/ϕ = 0.05, 0.10, 0.25 and 0.50. Calculations were also carried out for the straight bar e_o_/ϕ = 0. Calculations were performed for eight slendernesses of the bar expressed by the ratio of the distance between support points (ties) s and bar diameter s/ϕ = 5, 6, 7, 8, 9, 10, 12 and 15.

The Mander’s steel model [37] has been adopted, which is similar to the characteristics of σ-ε steel used in reinforced concrete structures (Figure 3d). The hardening curve in this model is described by the equation:

(1) $$\sigma_{s}{=f}_{\mathrm{su}}{+(f}_{\mathrm{sy}}-f_{\mathrm{su}}){\left(\frac{\varepsilon_{\mathrm{su}}-\varepsilon_{s}}{\varepsilon_{\mathrm{su}}-\varepsilon_{\mathrm{sh}}}\right)}^{p}$$

where

(2) $$p{=E}_{\mathrm{sh}}\frac{\varepsilon_{\mathrm{su}}-{\;\varepsilon }_{\mathrm{sh}}}{f_{\mathrm{su}}-f_{\mathrm{sy}}}$$

The strength parameters of steels are described as follows: yield strength f_sy_ = 400 MPa, tensile strength f_su_ = 600 MPa, hardening strain ε_sh_ = 0.010, ultimate strain ε_su_ = 0.10, Young’s modulus E_s_ = 200 GPa and initial hardening stiffness E_sh_ = 5 GPa.

In the numerical analysis of large elastic-plastic deformations of compressed bars, the COSMOS/M system will be used [38]. In the numerical model, the finite element BEAM2D with the option of plastic analysis was used, with the Huber-Mises-Hencky plasticity condition, associated with the flow law and isotropic reinforcement. Nonlinear material characteristics σ-ε with the PLASTIC option were introduced into the analysis. In the nonlinear analysis, the displacement control method was used, and the longitudinal displacement of the loaded end of the bar was treated as the displacement control parameter. The Newton–Raphson method was chosen as the solution method for nonlinear algebraic equations in consecutive incremental steps of the selected displacement parameter. The option of automatic step selection was used, modifying slightly the parameters of this option for individual examples. The aim of the calculations was to obtain the full load-displacement characteristics corresponding to the experimental compression test.

### 3.3. Results of the Analyses

**Figure 4:**
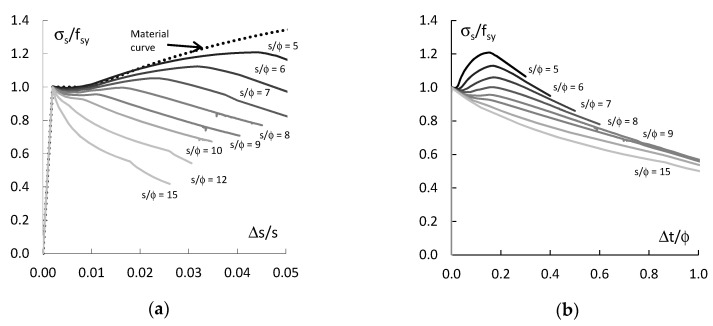
Behaviour of straight bars: (**a**) normalized stress versus average shortening and (**b**) normalized stress versus average lateral displacement.

Figure 4 shows the results of the analysis of the behaviour of the compressed straight bars e_o_/ϕ = 0.

These are the relations between the average stress in the bar σ_s_ = F/A_s_ normalized at yield strength f_sy_ (σ_s_/f_sy_) and the average strain of the bar ∆s/s (Figure 4a) and the average transverse strain ∆t/ϕ in the middle section of the bar (Figure 4b). As can be seen in the diagrams, the buckling resistance of the bars depends on their slenderness s/ϕ. The maximum stresses in the bars σ_s_ is greater than the yield strength f_sy_ if the bars slenderness s/ϕ < 8. For slenderness s/ϕ ≥ 8, the maximum stresses in the bars are equal to the yield strength. This is confirmed by the results of experimental tests of compression rebar [21], in which for slenderness s/ϕ < 8, the resistance of the rebar was equal to or greater than the yield strength.

The slenderness of the bars also has a significant influence on the lateral displacement of the bars’ centre section after buckling (Figure 4b). When the stresses in the bars does not exceed the yield point, the bars remain straight. However, when the stresses in the bars reach the yield point, the buckling of the bars occurs and they suffer lateral displacement. For slenderness of bars s/ϕ > 8, the increasing lateral displacements are accompanied by a progressive decrease in the load-bearing capacity, and then, their bending stiffness begins to determine the load-bearing capacity of the bars. However, when the slenderness of bars is sufficiently low s/ϕ < 8, the increase in lateral displacements is accompanied by an increase in the longitudinal force transmitted by the bars and the force transmitted by the bars decreases only when it reaches its maximum value greater than its yield point.

**Figure 5:**
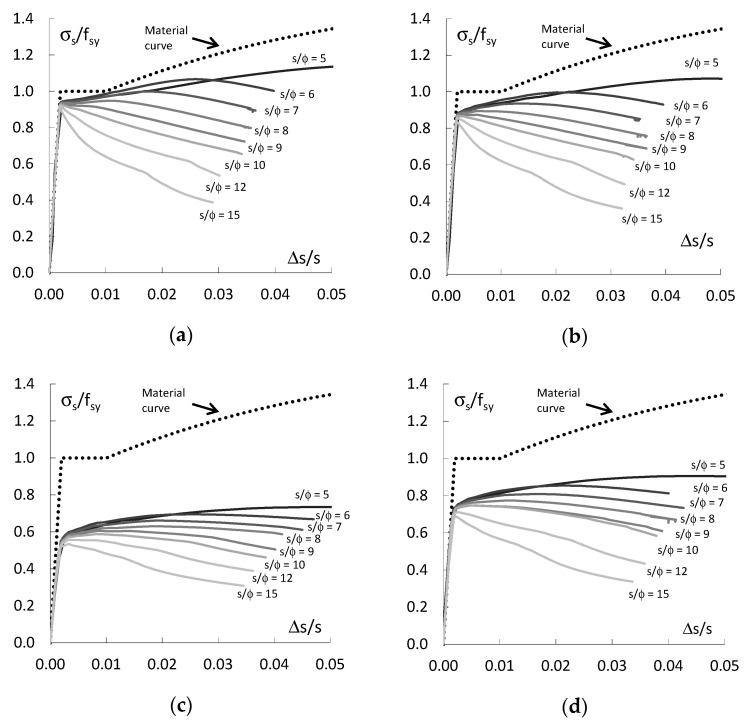
Normalized stress—strain curve for MD bars: (**a**) e/ϕ = 0.05, (**b**) e/ϕ = 0.10, (**c**) e/ϕ = 0.25 and (**d**) e/ϕ = 0.50.

Figure 5 shows the calculation results for the bending form (MD) of bars deformation (see Figure 2a and Figure 3a) with different imperfection amplitudes e_o_/ϕ and with different slenderness s/ϕ. Figure 5 illustrates the relationship between the average stress (σ_s_ = F/A) standardized with respect to the yield strength of f_sy_ and its average strain Δs/s. Figure 5 shows that the behaviour of bars depends very significantly on their au slenderness s/ϕ and the amplitude of imperfections e_o_/ϕ. The initial curvature of the bar makes the yield plateau disappear. The bars work elastically to a stress level below the yield point (σ_s_ < f_sy_). When the slenderness of the bars s/ϕ < 10 is exceeded, the load-bearing capacity of the bars increases very little regardless of the initial eccentricity of the longitudinal force e_o_/ϕ. If the imperfection amplitude e_o_/ϕ ≤ 0.10 and the slenderness of the bars s/ϕ ≤ 6, the resistance of the bars is greater than the yield strength. An increase in imperfection amplitude e_o_/ϕ reduces the resistance of bars with comparable slenderness.

**Figure 6:**
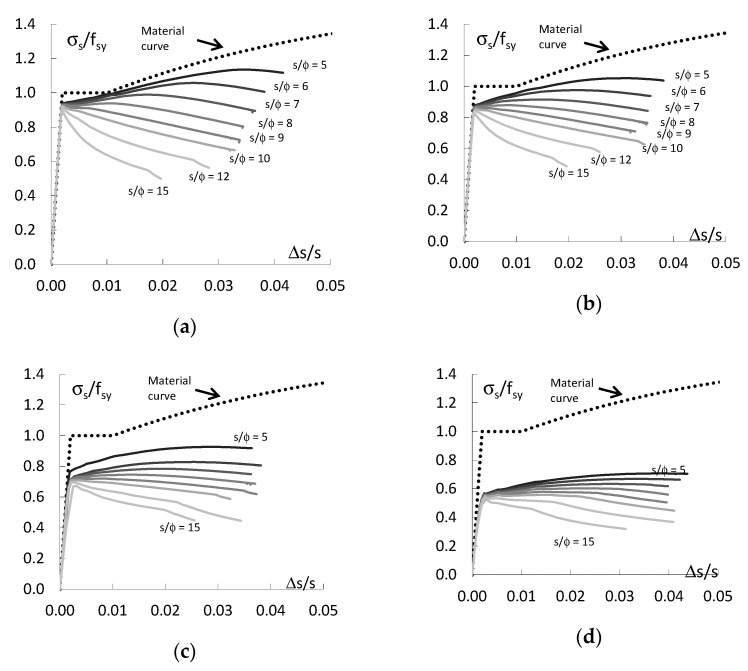
Normalized stress—strain curve for ND bars: (**a**) e/ϕ = 0.05, (**b**) e/ϕ =0.10, (**c**) e/ϕ = 0.25 and (**d**) e/ϕ = 0.50.

Figure 6 shows the calculation results for the compressive form (ND) of bars deformation (see Figure 2b and Figure 3b) with different initial curvatures e_o_/ϕ and different slendernesses s/ϕ. The behaviour of bars for compression deformation is very similar to that of bars with bending deformations. The behaviour of these bars also depends very much on their s/ϕ and imperfection amplitude e_o_/ϕ. The initial curvature of the bars causes the plastic flange to disappear and the bars work elastically to a certain load level below the yield point (σ_s_ < f_sy_). When the proportionality limit is exceeded, the resistance of the bars increases very little regardless of the initial longitudinal force eccentricity e_o_/ϕ if the bars slenderness s/ϕ < 10.

The resistance of the bars is greater than the yield strength if the initial eccentricity e_o_/ϕ ≤ 0.10 and the slenderness s/ϕ ≤ 6. An increase in the initial curvature of the bars e_o_/ϕ causes a decrease in the bars’ resistance regardless of its slenderness. The results of analyses of behaviour of ND bars are similar to those of experimental tests [21,27]. The differences are due to the different mechanical properties of the bars, which have an impact on the inelastic buckling of the bars [34]. This behaviour of the bars is also confirmed by the results of tests of confined RC columns [39], which showed that for sudden buckling of the reinforcement bars in the columns, there may even be enough elastic deformation.

**Figure 7:**
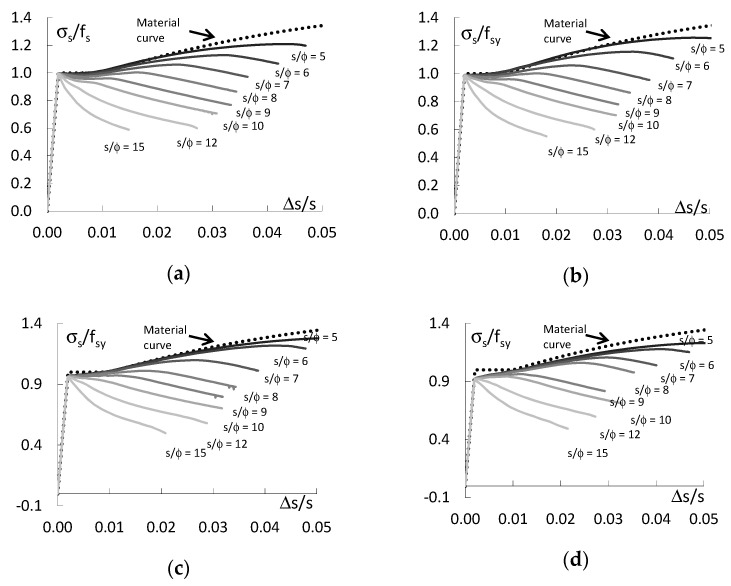
Normalized stress—strain curve for VD bars: (**a**) e/ϕ = 0.05, (**b**) e/ϕ = 0.10, (**c**) e/ϕ = 0.25 and (**d**) e/ϕ = 0.50.

Figure 7 illustrates the behaviour of the bars for shear deformation (VD) associated with the initial horizontal displacement of one of the supports (see Figure 2c and Figure 3c).

As can be seen, the behaviour of bars with shear deformations differs significantly from that of bars with bending and compression deformation shapes. The resistance of the bars in this case is much higher for all values of slenderness s/ϕ and initial curvature e_o_/ϕ than for the two previous forms of deformation. The initial transverse displacement of the supports has little effect on the resistance of the bars, and the behaviour of the bars is almost the same as that of straight bars. The resistance of the deformed bars is comparable to that of the straight bars (see Figure 4). It is only for imperfections e_o_/ϕ = 0.25 and 0.50 that a difference is noticed, i.e., the limit of proportionality is less than the yield strength (σ_s_/f_sy_ < 1). Regardless of the amplitude of the imperfections e_o_/ϕ, the strength of the bars is higher than the yield strength if the slenderness s/ϕ < 9.

While the results of numerical analyses of ND bars are confirmed by experimental studies [21,27], unfortunately, there are no results available for experimental studies of MD and VD bars. However, it can be assumed that since there is a correspondence between the numerical analysis and the experimental results for ND bars, this is also the case for MD and VD bars.

### 3.4. Analysis and Summary of Results

**Figure 8:**
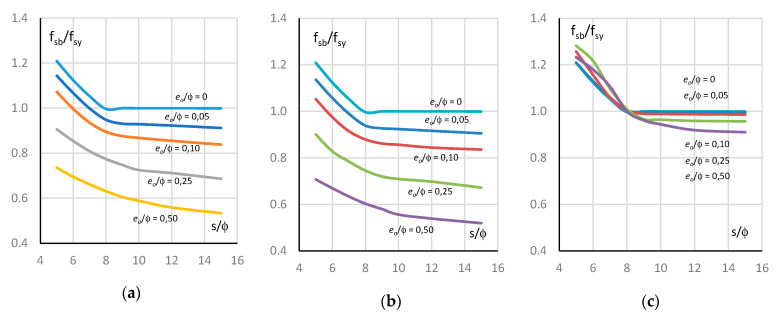
Normalized buckling resistance—slenderness curve for: (**a**) bending deformation (MD), (**b**) compression deformation (ND) and (**c**) shear deformation (VD).

Figure 8 shows the diagrams illustrating the effect of the slenderness of bars on their buckling resistance for the three initial shapes of deformation. For comparison, the figure also shows diagrams for straight bars. These are graphs of the relationship between the normalized buckling resistance f_sb_/f_sy_ and the slenderness of the bars s/ϕ. The buckling resistance of the bars corresponds to the maximum average stress in the bars f_sb_ = σ_s,max_. The buckling resistance f_sf_ of straight bars e_o_/ϕ = 0 is greater than the yield strength f_sy_ if their slenderness s/ϕ < 8. For slenderness s/ϕ = 5, the buckling resistance is 20% greater than the yield strength f_sy_. For slenderness s/ϕ ≥ 8, the yield strength of straight rebar is f_sb_ = f_sy_.

Geometric imperfections for the bending (Figure 8a) and compression (Figure 8b) shapes of the bars deformations result in a significant reduction of buckling resistance compared to straight bars. Further, there is a decrease in the ability of the bars to maintain resistance as the slenderness of the bars and amplitude imperfections increase. The variations in the buckling resistance of bars with bending deformations and compression deformations are similar, with the reductions in buckling resistance for compression deformations being slightly higher than for bending deformations. The resistance of bars with bending and compression deformations is greater than the yield strength if the initial eccentricity e_o_/ϕ ≤ 0.10 and the slenderness s/ϕ ≤ 6. In other cases, the bars resistance is less than the yield strength. In extreme cases, the deviation from the straight form of the bars (e_o_/ϕ = 0.50) results in a nearly 50% decrease in the resistance compared to the straight form of the bars. For imperfection amplitude e_o_/ϕ = 0.25, this decrease is about 30%; for imperfection amplitude e_o_/ϕ = 0.10, this decrease is about 15% and for imperfection amplitude e_o_/ϕ = 0.05, this decrease is about 10%.

The behaviour of bars with initial shear deformations is surprising and completely different from the behaviour of bars with bending and compression deformations (Figure 8c). In this case, the behaviour of the bars is very similar to that of straight bars. The buckling resistance of such deformed bars is higher than the yield strength and, at the same time, higher than the resistance of straight bars if the slenderness s/ϕ < 8. If the slenderness of the bars s/ϕ > 8, the resistance of imperfect bars is lower than the yield strength and the resistance of straight bars. This regularity is observed for all amplitudes of imperfections. The buckling resistance of deformed and straight bars is equal to the yield strength for slenderness s/ϕ = 8.

**Figure 9:**
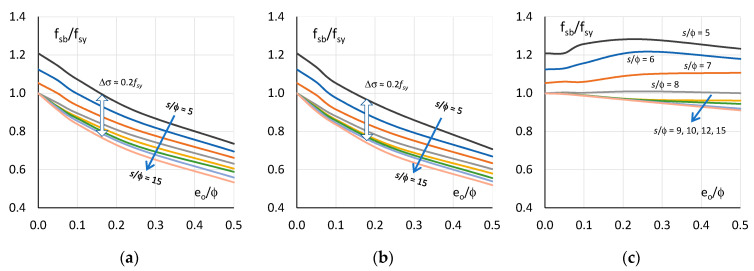
Normalized buckling resistance—amplitude of imperfection curve for: (**a**) bending deformation (MD), (**b**) compression deformation (ND) and (**c**) shear deformation (VD).

Figure 9 illustrates the effect of the amplitude of geometric imperfections on the buckling resistance of the bars. For bending deformations (Figure 9a) and compression deformations (Figure 9b), the effect of the imperfection amplitude on the bars’ behaviour is very similar. The differences in the changes of bars resistances are small. In both cases, the resistance of the bars decreases with an increase in the imperfection amplitude for all bar’s slenderness, but the decreases in resistance are slightly higher for compression deformations. It can also be seen that for bending and compression deformations, the decrease of their resistance in the analysed range of imperfection and slenderness amplitudes is constant. The difference in load capacity between the bars with the smallest and the largest slenderness is approximately 20% of the yield strength for the whole range of imperfection amplitudes. For the highest imperfection amplitude e_o_/ϕ = 0.5, the resistance of the bars is between 50% and 70% of the yield strength depending on the slenderness of the rebar. For the remaining imperfection amplitudes, the buckling resistance of the bars is proportionally higher.

The behaviour of bars with shear deformations is different (Figure 9c). The load capacity of deformed bars with slenderness s/ϕ = 5, 6 and 7 is greater than the yield strength and, surprisingly, the load capacity of these bars increases with increasing imperfection amplitude and is greater than the load capacity of straight bars e_o_/ϕ = 0. If the slenderness of the bars s/ϕ = 8, then the load capacity of deformed bars is equal to the yield limit and is the same as the load capacity of straight bars. If the slenderness of the bars s/ϕ > 8, then their load capacity decreases slightly and remains at a level not exceeding 0.9 f_sy_.

**Figure 10:**
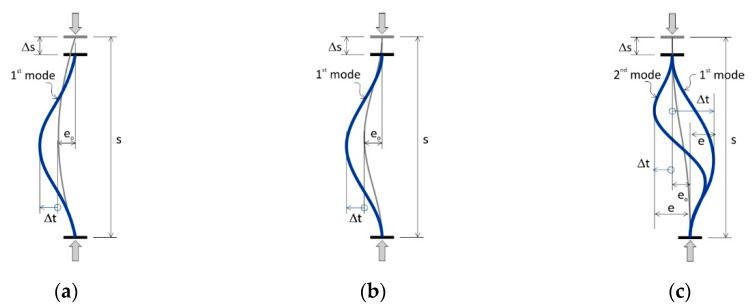
Mode of bars buckling: (**a**) bending deformation (MD), (**b**) compression deformation (ND) and (**c**) shear deformation (VD).

The previously described behaviour of predeformed compression bars can be explained by analysing the mode of their buckling. This is illustrated in Figure 10.

As mentioned above, the buckling resistance of bar with bending deformations (Figure 10a) is slightly higher than that of bars with compression deformations (Figure 10b). As can be seen in these figures, for bars with bending deformations, their buckling mode does not match the shape of the initial deformations, and for bars with compression deformations, their buckling mode follows the shape of the initial deformations, hence the higher resistance of the former. Such a phenomenon is called “coupled buckling” [40]. For bars with shear deformations, two forms of modes of buckling were noted (Figure 10c). These can be explained by the unexpectedly high buckling resistance of these bars compared to bars with other deformation shapes. Slenderness bars s/ϕ = 5 and 6 had the second mode of buckling, while slenderness bars s/ϕ ≥ 8 had the first mode of buckling, whereas slenderness bars s/ϕ = 7 initially had the second mode of buckling and then, transitioned into the first mode of buckling (Figure 10). The buckling resistance for the second mode of buckling is greater than that of the same member for the first mode of buckling. In this case, the longitudinal force acted on the eccentricity e = e_o_ + ∆t increasing with the shortening of the bar ∆s. For greater slenderness s/ϕ ≥ 8, horizontal displacements ∆t was the opposite sign, so the eccentricity e = e_o_ − ∆t decreased to zero and then after changing the sign, the eccentricity increased forcing the first mode of buckling. Therefore, the bending effect caused by the eccentric action of the longitudinal force was smaller than in the case of the initial deformation of bending and compression. Hence, the higher buckling resistance of bars with shear deformations was observed.

## 4. Conclusions

The longitudinal reinforcement bars in reinforced concrete columns are deformed by the acting loads. This has a significant impact on their behaviour under compressive forces. The numerical analyses carried out showed that the following three factors have an influence on the inelastic buckling of bars: the shape of initial deformation, the amplitude of geometric imperfection and the slenderness of the bars. The buckling resistance of the bars and their ductility both depend on these three parameters.

The behaviour of compression bars with bending MD and compression ND shape of initial deformations is almost identical, but it differs significantly from the behaviour of bars with shear shape of deformation VD. The load capacity and ductility of VD bars are similar to the capacity and ductility of straight bars. The decrease in the load capacity of bars with the form of VD buckling for the entire range of analysed imperfections is very small compared to the load capacity of bars without imperfections. This amounts to a maximum of 5% for imperfections e_o_/ϕ = 0.50. In contrast, the loads capacity of MD and ND bars is high: the load capacity of these bars is lower than the load capacity of bars with a typical VD buckling from 5% to 40% depending on the size of the imperfection.

The influence of imperfection amplitude on the buckling resistance of bars also depends on the shape of the initial deformations. The load capacity of bars with bending and compressed shapes of initial deformations decreases with increasing imperfection amplitudes. Regardless of the slenderness of the bars and the amplitude of imperfection, their load capacity is 50–60% of the load capacity of straight bars. In the case of bars with a shear form of deformation, the load capacity of these bars for small slenderness s/ϕ = 5–7 increases with the amplitude of imperfection and is greater than the load capacity of straight bars by 5–10%. For bars with a greater slenderness s/ϕ > 8, the load capacity of the bars decreases slightly and does not exceed 10% of the load capacity of straight bars.

As the slenderness of the bars increases, the buckling capacity of the bars decreases: bars with a smaller slenderness (s/ϕ < 8–10) lose more of their load capacity. Bars with a larger slenderness, however, have a smaller decrease in the loss of load capacity. The load capacity of straight bars is at least equal to the yield strength, and if the slenderness of the bars is s/ϕ < 8, the load capacity of the bars is greater than the yield strength.

The stirrup/tie spacing in beams and columns in seismic-resistant frames shall not exceed eight diameters of longitudinal reinforcement bars, and in order to take account of geometric imperfections, the load capacity of the bars shall be reduced by 40%.
